# Factors that enable or limit the sustained use of improved firewood cookstoves: Qualitative findings eight years after an intervention in rural Mexico

**DOI:** 10.1371/journal.pone.0193238

**Published:** 2018-02-21

**Authors:** Minerva Catalán-Vázquez, Rosario Fernández-Plata, David Martínez-Briseño, Blanca Pelcastre-Villafuerte, Horacio Riojas-Rodríguez, Laura Suárez-González, Rogelio Pérez-Padilla, Astrid Schilmann

**Affiliations:** 1 Clinical Epidemiology Department, National Institute of Respiratory Diseases, Mexico City, Mexico; 2 Epidemiology and Social Sciences in Health Department, National Institute of Respiratory Diseases, Mexico City, Mexico; 3 Centre for Health Systems Research, National Institute of Public Health, Cuernavaca, Morelos, Mexico; 4 Environmental Health Department, Centre for Population Health Research, National Institute of Public Health, Cuernavaca, Morelos, Mexico; 5 Tobacco and COPD Department, National Institute of Respiratory Diseases, Mexico City, Mexico; University of Miami, UNITED STATES

## Abstract

**Objective:**

The aim of this study was to analyze the factors enabling/limiting the use of improved cookstoves among rural fuel wood users from one mestizo and two indigenous communities eight years after an intervention in the state of Michoacan, in Mexico.

**Methods:**

A qualitative study with an ethnographic perspective was conducted in 2013/2014 based on 62 interviews with women who had participated in an improved firewood cookstove program in 2005. Thematic qualitative content analysis was performed.

**Results:**

Very few women from the indigenous communities were using the improved cookstove at the time of the study; the majority had dismantled or had ceased using it; whereas most of those from the mestizo community were using it for all of their cooking activities. In the indigenous communities, characterized by extended families, uptake of new technology was limited by traditional routine practices, rearrangement of rooms in the house, attachment to the traditional stove, a low- or non-risk perception of woodsmoke; gender relations, insufficient training, non-compliance with program recommendations and design-related aspects. Conversely, in the mestizo community, the uptake of the improved cookstove was favored by routine cooking practices in a nuclear family, a previous use of a raised cookstove and social representations on the health-disease-death effects of woodsmoke vs. the health benefits of cooking with improved stoves. The sociocultural dimension of communities and the cookstove design are aspects that either favor or limit the use of improved cookstoves in indigenous and mestizo populations.

**Conclusions:**

Effective cookstove programs must take these elements into account from their early planning stages, and blend them into implementation and follow-up. Project communication, training and differentiated follow-up activities ensuring the operation and maintenance of the cookstove, should be designed according to the specific needs and traditions of each community; they should be based on the preferences and needs of the users.

## Introduction

### Solid fuel use and cooking technologies

Solid fuels are used extensively for cooking and home heating in developing countries, especially in rural areas. According to recent estimates, in Mexico, there are 16.8 million exclusive fuelwood users and 5.8 million mixed users (around 20% of the population) mainly in the rural and indigenous areas [1]. The traditional use of solid fuels in inefficient devices results in excess fuel use and the release of products of incomplete combustion. Health impacts are driven by indoor exposures mainly during cooking, which disproportionately affect women and small children [2]. There has been a global focus on improved cookstoves (ICSs) and clean fuels based on their potential benefits in three areas: health of household members, local environmental quality, and regional climate benefits. However, ICS and clean fuel dissemination programs have presented low uptake rates [3–5]. Introduction of new cooking fuels and stoves in many areas has been best described as a “stacking” process: New stoves and fuels do not usually substitute 100% for traditional ones, but rather are initially used for certain cooking tasks. An in depth understanding of the household energy use and how the new stoves and fuels can displace older devices across all household energy tasks is critical to achieve the expected health benefits by such programs [6–8].

The kitchen is a complex social space that is both critical to the material well-being of the household and imbued with deep cultural meaning, which raises a unique set of characteristics in the intersection of the culture, environment, and fuel access [4, 7–11]. The stacking has strong implications in terms of the long-term impacts of interventions [8], precludes adequate reduction in exposure levels [12] and therefore the health benefits have been less than expected.

The World Health Organization published indoor air quality guidelines regarding household fuel combustion which include recommendations on emission rate targets from household energy fuels and technologies that pose minimal health risks [13]. Few households worldwide can achieve the air quality standards without displacing the polluting traditional open fire with modern technologies [8, 14].

Studies across the world have identified a wide variety of contextual factors which influence the process of ICS uptake. For instance, after reviewing 137 ICS programs in 47 developing countries, the World Bank reported that efforts tend to fail [15] if any of the following conditions apply: cookstoves and fuels cannot be purchased or easily obtained; those who determine that the ICS is required are outside “experts”, the ICSs are provided as part of a technical package without taking user preferences into account, the design does not resemble that of traditional stoves, the ICS is difficult to light and requires pellets, and finally, monitoring and evaluation are not designed and budgeted as part of the program. More recent studies have identified the following social determinants increasing the uptake processes: higher income and schooling [9, 16–18], fuelwood price, household size and credit access [16], house ownership [9], previously owned and used LPG stove [10], exposure to messages about the benefits of improved cookstoves [18] and the agreement between the technical characteristics of the stove with the social and cultural needs and expectations [9, 16–19].

Over the past 30 years, the international community has gradually shifted the focus of analysis towards the sociocultural contexts of users. Several studies have explored the sociocultural factors related to the user decision to choose a new cooking technology or to fulfill other household energy needs. Due to its importance, these issues are still under research. As mentioned by Pine et al (2011) [20], “many programs to promote these improved technologies have failed in the long run because they did not take variations in cultural preferences, local cooking needs, patterns of household fuel use, and other social and economic factors into account”. Some of the studies under this perspective have been carried out in Guatemala [21]; in Mexico [4, 22]; in Mozambique [23]; in Bangladesh [24]; in Kenya [25][26]; in India [27]; in Mongolia [28]; and in Sudan [18]. Some other studies have explored the influence of gender on the adoption and use of ICS [29, 30].

The study in Sudan indicated that traditional ways of cooking cover a large number of sociocultural functions and practices that have not been considered by ICS programs [18]. In addition, a systematic review study showed that indigenous and other regional ethnic groups are not likely to adopt clean fuel and ICSs because of the intrinsic value that traditional cooking techniques represent [16]. Similarly, a study in Peru, Nepal and Kenya analyzing contributing factors for rendering ICS interventions more effective pointed out that both general similarities and locally distinct practices and norms can be identified in traditional stove usage across regions. Traditional stoves accommodate not only specific cooking styles, but also fuel types and resources available for their maintenance and renovation. They allow users to cook their local dishes and repair their stoves easily. The authors concluded that ICS designs need to take into account the diversity of values and necessities of potential users; as a consequence, a single design for different regions is not advisable because it cannot cover diverse cooking practices and other household energy needs, or respond to local resource restrictions [31]. In this context, stacking becomes important, given that each combination of technologies and fuels covers a niche of specific tasks according to the needs and preferences of the users [7].

After analyzing the factors behind ICS uptake in Africa, Asia, Central America and the Caribbean [15, 19], studies have concluded that programs incorporating the needs and preferences of users are more likely to be successful. According to these studies [19], women attach greater value to features other than fuel savings (e.g., the time stoves take to warm up, aesthetics and the social status of possessing improved technology). ICSs must therefore be competitive vis-a-vis the traditional ones in numerous ways (i.e., ease of operation, safety and time savings) for users to clearly perceive their benefits. Few studies have adopted a user’s perspective to analyze the factors in the selection among different cooking technologies [32], nor have they followed up on ICS implementation to investigate sustained usage and to identify the factors underlying the uptake process.

### Studies in Mexico

A study on fuelwood use and management strategies in the Altos de Chiapas region, showed how sociocultural, economic, climatic and technical factors pose barriers to the acceptance of technological changes [33].

The rural household energy program with the development and dissemination of the ICS Patsari is a well-documented project in Mexico [34]. The Patsari stove is an efficient multipot wood-burning stove built in situ with the help of a mold and local materials, including a big flat pan or comal, a chimney and/or two secondary pots. The size and inner dimensions of the stove were optimized to produce a cleaner combustion. The implementation of the program in this case included the installation of the ICS and an initial training with a short follow up during one year as part of the study, but the supply chain for spare parts was not assured and people had to rely on themselves for stove operation and maintenance [17].

Use of the Patsari cookstove in actual field conditions, has been shown to achieve average reductions of 70% in indoor air pollution concentrations [35, 36], of 56% in household fuel consumption [37], and of 74% in greenhouse gas emissions [38] compared with open fires. A health impact assessment showed that the actual use of the Patsari stove, as reported during field worker visits, was significantly associated with a reduction of symptoms related to wood smoke and of lung function decline over 1 year of follow-up in women [5] and a protective effect mainly on the upper and lower respiratory infection duration in children [39]. It has been documented that the processes of ICS uptake are driven by both household and community components [20]. The adoption of a cookstove should not be regarded as an isolated event, but rather as a complex process where cooking practices depend on specific user-technology-fuel interactions [40]. From the perspective of users in the Purepecha Meseta region, it is clear that these processes are permeated by social, cultural and environmental issues [4].

In order to better understand the individual and sociocultural factors underlying the adoption and sustained use of ICSs in the Mexican Purepecha region, the aim of this study was to analyze the factors enabling/limiting the use of improved cookstoves among rural fuel wood users from one mestizo and two indigenous communities eight years after an intervention in the state of Michoacan, in Mexico.

## Methods

### Study population

The Purepecha Meseta region of the Mexican state of Michoacan, sitting over 2,400 meters above sea level, is dominated by pine and oak forests with a temperate weather with large diurnal variation, particularly in winter. The household solid fuel use is highly prevalent and there is a seasonal space heating need. The access and use of LPG is low. The region is inhabited by Purepecha indigenous communities of pre-Hispanic origin, and more recently settled mestizo rural communities [41].

A randomized community trial was conducted in six communities, from the Purepecha region to evaluate the impact of a Patsari ICS program implementation on women and children health. Among 889 households complying with the inclusion criteria (using open wood fires and having a child under 5 years old), 668 agreed to participate and were randomized to receive the stove at the beginning (early 2005) or end (mid 2006) of the study [5, 39]. Maximum ICS utilization (around 70%) was documented four months after installation and then declined steadily from the eight month (55%) onwards [20]. A follow up study was conducted among the cohort of rural women in four communities established during this community trial in 2012/13 to assess health outcomes (lung function) and learn about the household fuel use during the period. Two communities were excluded from this follow up because of logistic issues and insecurity. From a total of 257 participants in 2012, only 15.5% (n = 40) of the women still used the Patsari ICS, 15.5% (n = 40) still possessed it but did not use it, and 69% (n = 177) had dismantled it [42]. A subsample of participants was selected from three communities to participate in the qualitative study presented here.

### Study design

We used a qualitative design with an ethnographic perspective based on semi-structured interviews with the adult women participating in the Patsari follow-up study [42]. Our report was formulated according to the qualitative research reporting criteria [43]. Our selection of an ethnographic theoretical perspective arose from our interest in the behavior and perceptions of the social actors related with the adoption and use of the ICS in their particular sociocultural context. Our study sought answers to the following questions: What factors enabled/limited ICS adoption and use? And specifically with regard to the Patsari participants who had not adopted or were not using the ICS, why did they reject the new technology? We used semi-structured interviews because they allowed for understanding how the women perceived their own practices and experiences as well as the significance they attributed to adopting and utilizing an ICS in their homes. The study was approved by the Science and Bioethics Committee of the National Institute of Respiratory Diseases of Mexico (INER by its Spanish initials) under protocol number S02-14 as a minimal risk study with an oral informed consent for the interviews and its audio recording.

### Selection of participants

**Fig 1:**
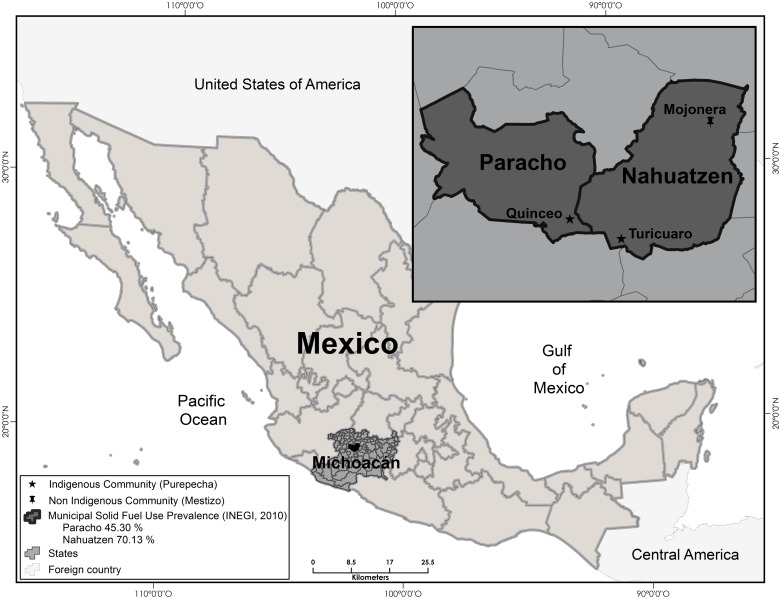
Map of the three participating rural communities located in the Mexican state of Michoacan.

The interviews were conducted in three communities—one mestizo (La Mojonera) and two indigenous (Quinceo and Turicuaro)—of the Mexican Purepecha region (Fig 1). Participants were selected by means of snowball sampling from the Patsari follow-up study cohort [42].

As part of the Patsari follow-up study, our participants were classified according to the status of the ICSs installed in their communities in 2005 and 2006, namely whether they were in use, disused or nonexistent (dismantled). The number of interviews was determined according to the theoretical saturation criterion; that is, interviews were suspended when they ceased to provide new elements for a more in-depth understanding of the object of our study [44]. A total of 62 interviews were carried out: 15 from Quinceo, 22 from Turicuaro and 25 from La Mojonera. The participants were personally invited at their homes. The rural health assistant in the two indigenous communities served as the link between the researcher and study participants; at times, she also served as interpreter, since the women spoke the Purepecha language. In the mestizo community, the nurse at the health center assisted in contacting participants but was not present during the interviews.

### Piloting

We designed the interview guide on the basis of a literature review focused on the topic of our study, placing special emphasis on the sociocultural characteristics of our sample population such as its attachment to traditional cooking technologies and practices. The interview script was tested on a group of four women from Quinceo and was subsequently redesigned as regards the introduction, sequence of its items and duration of the interviews.

### Procedure

First, we presented the study to the local, civil, agrarian and health authorities. Subsequently, we carried out three field trips across the study communities in order to become acquainted with the residents, observe their daily routines and locate the homes of potential participants. Two visits were made to each prospect: the first to present the study, the purposes and approximate duration of the interview, the confidentiality of the information provided and the most convenient timing for the interview. Those who expressed an interest in participating were then asked to give their oral informed consent to the interview and its audio recording. During the second visit, we conducted the interview and inquired about housing conditions, including the kitchen characteristics. All interviews were carried out by the principal investigator (MCV), a researcher with experience in qualitative studies of women from indigenous and marginalized communities, who had participated in the Patsari follow-up study but not established contact with the interviewees. Conducted face to face in the homes of the women, the interviews lasted approximately 40 minutes each and ran from September 2013 to April 2014. The rural health assistant in the two indigenous communities served as interpreter when the participating women did not speak Spanish fluently.

In order to understand the factors influencing women’s decision to adopt and utilize the ICS, we explored a combination of sociocultural determinants and characteristics including gender, social class, ethnicity, age group, the mother-wife condition, knowledge, customs and traditions [32, 45]. Our interview script identified four relevant topics: (1) family and everyday life, especially the type and composition of family, the occupation and health of its members and various aspects of women’s everyday lives; (2) social representations on being a woman, specifically, community images of what a woman should be, including the knowledge that mothers transfer to their daughters; (3) technologies used for cooking and other domestic tasks. We asked respondents at what age they had learned how to use the traditional open fire, what domestic tasks they used it for and what advantages and/or disadvantages they attributed to traditional technology. We asked also what the history of the ICS stove was and whether or not they still kept and used it. Those who answered positively were asked what tasks they used it for and what advantages and/or problems derived from its use. Those who answered negatively were asked to explain why they had abandoned the stove; and (4) health risks attributed to woodsmoke and the effects, if any, that the respondent, family members or community residents had experienced as a result of inhaling woodsmoke from the traditional open fire.

We conducted the interviews mainly in the kitchen, some of them while the respondent performed one of her cooking activities. We recorded the information in a notebook adapted as an ethnographic register and audiotaped the conversation. The sessions occurred without the presence of other researchers and only occasionally in the company of a family member. During the interview visit, we registered the location of the kitchen (in/outside the dwelling), its type of construction (traditional/modern), the status and location of the Patsari ICS, and the location of the traditional open fire and other furniture. All of these elements provided useful information for the interpretation of the study results, particularly those related to cooking technologies.

### Data analysis

We transcribed the audios using a word processor designed for professionals in the INER Epidemiology and Social Science Research Department, with transcriptions verified by MCV. Based on the method proposed by Taylor & Bogdan [46], data analysis was performed along the following stages: (1) repeated readings of transcripts; (2) definition and elaboration of a codebook according to the categories in the interview guide themes; (3) identification of emerging themes; (4) coding of linguistic material using Atlas-ti software (versions 5.2); and (5) data editing, description and analysis. Coding of texts was defined according to an initial draft which was progressively refined through discussion and consensus.

## Results

### Characteristics of the study communities

Of pre-Hispanic origin, the indigenous communities of Turicuaro and Quinceo lie in the municipalities of Nahuatzen and Paracho, respectively. La Mojonera, a mestizo community formed after the Mexican Revolution, lies in the Nahuatzen municipality, with its cooperative land tenure established in 1934 [41]. Table 1 illustrates the demographic, economic, educational and household characteristics of the three sample communities. La Mojonera presented the lowest number of illiterate women and the economically active population was integrated by 11–13% of its female population. The three sites shared the same proportion (roughly 14%) of female-headed households. Dwellings with dirt floors were found throughout the three sites but prevailed in the indigenous communities: 48% in Turicuaro and 65% in Quinceo. La Mojonera showed a lower prevalence of piped water (36%) than the other two communities [47].

**Table 1:** Profile of the study communities according to the 2010 National Census.

Variable	La Mojonera (%)	Turicuaro (%)	Quinceo (%)
**Population**			
Total (number of people)	1,403	3,388	2,692
Female	51.7	50.6	51.1
**Economic aspects**			
Main economic activity: Timber extraction and wood industry	-	Small-scale timber mills	Small-scale timber mills
Economically active female population^§^	12.3	11.1	13.2
**Indigenous language and education**			
Female population speaking an indigenous language^¶^	0.3	95.6	98.1
Illiterate female population 15 years and older	11.3	50.7	41.6
Female population 18 years and older with post-basic education	12.1	4.7	5.5
**Health and marital status**			
Population without health-care coverage	42.6	67.6	24.9
Population 12 years and older who were married or in a common-law partnership	62.6	64.0	62.1
**Home and household characteristics**			
Female-headed households	14.2	14.2	15.7
Households with dirt floor	29.8	48.0	65.0
Households with a single room	1.4	9.4	8.6
Households with piped water	36.1	87.9	94.1

^§^Women 12 years and older who were working, had work but were not working, or were seeking work during the reference week

^¶^Women aged 3–130 years speaking an indigenous language

Information from the 2010 National Census available at http://www.inegi.org.mx/est/contenidos/proyectos/ccpv/cpv2010/

### Participant characteristics and household fuel use

Table 2 illustrates the characteristics of the study population groups and their ICS and other cooking devices status. On average, the women in the indigenous communities were younger (33.1 vs. 36.5 years) and had two years less schooling (a total of 4.5 years) than those in the mestizo community. Practically all of the women in the study population were married. The indigenous communities obtained their water mainly from a well, whereas La Mojonera used jug water.

**Table 2:** Characteristics of participants by ethnic group.

Variable	La Mojonera (n = 25) n (%)	Turicuaro/Quinceo (n = 37) n (%)
Age [mean (SD), years]	36.5 (5.7)	33.1 (6.2)
Purepecha ethnicity	0 (0)	37 (100)
Schooling [mean (SD), years]	6.8 (2.6)	4.5 (3.3)
**Marital status**		
Married	24 (96.0)	36 (97.3)
In unión	0 (0)	1 (2.7)
Single	1 (4.0)	0 (0)
**Family type**		
Nuclear	21 (84.0)	17 (46.0)
Extended	4 (16.0)	20 (54.0)
Number of extended families sharing one kitchen	0 (0)	16 (43.2)
**Kitchen characteristics**		
Tin roof	22 (88.0)	34 (91.9)
Dirt floor	6 (25.0)	11 (31.4)
Brick or wooden walls	14 (60.9)	24 (72.7)
Separate from the house	19 (79.2)	30 (88.2)
Electricity	25 (100)	37 (100)
**Drinking wáter**		
Well	3 (13.0)	15 (42.9)
Water truck	1 (4.3)	1 (2.9)
Water jug	9 (39.1)	12 (34.2)
Piped	10 (43.6)	7 (20.0)
**Daily cooking time**[mean (SD), hours]	3.6 (1.7)	3.7 (1.4)
**Status of the Patsari stove installed under the study**		
In use	23 (92.0)	9 (24.3)
Abandoned	2 (8.0)	6 (16.2)
Nonexistent (dismantled)	0 (0.0)	22 (59.5)
Presence of an open fire	8 (32.0)	37 (100)
Presence of a LPG stove	8 (32.0)	4 (10.8)

In all three communities, kitchens of planks and gabled tin roofs were mainly built separately from the other rooms around a common patio. Previous publications on our study contain photographs of the typical described household and kitchen characteristics [5, 35]. The “Piso firme” Program, a large-scale government initiative for replacing dirt floors by cement floors, was implemented after the introduction of the Patsari cookstoves in this region.

**Fig 2:**
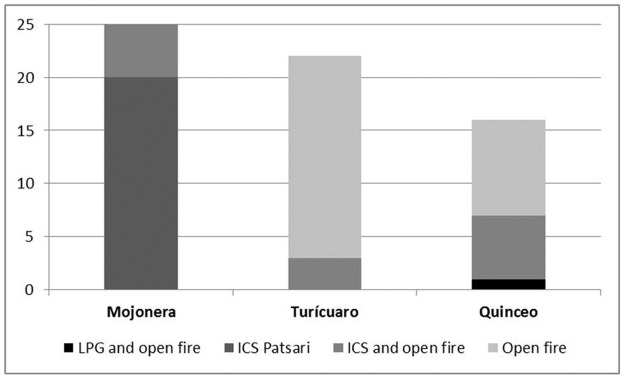
Household fuel use on a regular basis by study community (number of women in each category).

As shown in Table 2 and Fig 2, a traditional open fire, generally U shaped and built at floor level with overlapping bricks laid in the center of all indigenous kitchens. Twenty eight of the indigenous women still cooked exclusively on the traditional open fire; 22 of them had dismantled the ICS and in 6 cases the stove was abandoned, while the other 9 had the ICS in use combined with the open fire.

In contrast, the ICS was present in the entire mestizo kitchens; 23 women were using only the ICS and 2 women combined it with an open fire for some cooking tasks. LPG stoves were present in 4 indigenous kitchens, but only one woman used it regularly in combination with the open fire. Although the LPG stove was present in 8 kitchens from La Mojonera, none of the women used it regularly.

### Contextual use of the ICS (families and their everyday activities)

Twenty of our 37 Purepecha participants belonged to extended families including as many as 13 individuals from different generations. In 16 of the 20 interviews, we noted that the women cooked together. Although the nuclear families in the indigenous communities performed their daily cooking in their own homes, they joined their maternal or paternal families to cook for their frequent celebrations and social events. The husbands of the indigenous women were involved in agriculture or the wood industry or worked in a trade as masons or musicians.

By contrast, the mestizo community was composed mostly of nuclear families (n = 21) where women joined in family cooking activities on only four occasions. Most of the husbands of respondents were engaged in agriculture (cultivating oats and corn) while the rest worked at the community sawmill or took temporary jobs.

As part of their everyday activities, the indigenous women (1) **prepared meals**: took the *nixtamal* (corn cooked in limewater and then ground to make dough for the *tortillas*) to the mill in the morning and afternoon, made *tortillas* once or twice a day, prepared breakfast and lunch, served the family meals, took their husbands a snack in the cornfields or wood sites, and prepared the *nixtamal* for the following day; (2) **did housework**: swept the kitchen and patio floors, washed the dishes and cooking utensils and washed and ironed clothes for the entire family; (3) **made handicrafts**: weaved or knitted traditional clothing such as jackets, blouses, skirts and shawls as well as napkins for their own use or for sale in/outside the community; and (4) **worked on the farm fields**: sowed, cleaned, harvested the crops and collected firewood. Respondents related that gathering firewood from the fields was a pleasant activity because it drew them out of their everyday routine and allowed them the opportunity to walk and do exercise.

The mestizo women performed the same activities as their indigenous counterparts, except they made *tortillas* only twice or three times weekly. Some respondents even reported making *tortillas* for the entire week, either because their families were small or simply because they had grown tired of making *tortillas* day after day. They also reported not making handicrafts or having to go to the fields for firewood because they obtained their supply from the community sawmill.

Both in the indigenous and mestizo communities, the women agreed that of all their daily tasks, doing the laundry for the entire family was the most burdensome because of the physical effort and time required to wash such a large amount of clothes by hand.

“*The heaviest work is doing the laundry, …yeah, it’s really heavy. I get more tired from washing clothes; no, the rest isn’t that tiring, -as for making tortillas- no, because I make two and a half kilos of dough. I knead it with my own hands.*”

*(49-year-old female, ICS in use, Quinceo)*.

Preparing a large number of *tortillas* for an extended family, even a couple times a day, is not perceived as a heavy task, which can be a barrier to change the practice of this activity using the traditional open fire.

### Enablers and barriers for ICS use

Six themes relevant to ICS use emerged during data analysis: (1) the social representation on the role of women preparing *nixtamal* and *tortillas* on the traditional open fire; (2) the uses, advantages and disadvantages of the traditional stove; (3) the uses and advantages of the ICS; (4) reasons for abandoning the ICS; (5) reasons for not keeping the ICS; and (6) woodsmoke as a health risk. Table 3 summarizes the themes and subthemes identified during thematic analysis, classifying them as enablers or barriers for the sustained use of ICSs by ethnic group.

**Table 3:** Enablers and barriers for sustained use of the ICS by ethnic group.

Analysis topics	Indigenous communities		Mestizo community	
	Barrier	Enabler	Barrier	Enabler
**Women, the traditional open fire, *nixtamal* and *tortillas* (gender role)**				
Know how to use the traditional open fire.	**✔**			**✔**
Prepare *nixtamal*.	**✔**			**✔**
Make *tortillas* as much as twice a day.	**✔**			
**Traditional open fire: uses, advantages and disadvantages**				
Allows for preparing large amounts of food and *tortillas* during sowing and harvesting seasons.	**✔**			
Allows for preparing large amounts of food and *tortillas* for special celebrations.	**✔**			
Is fast and practical, uses all types of firewood (green branches, all sizes).	**✔**			
Heats up the house.	**✔**			
Fire can be directly observed.	**✔**			
Represents a vital kitchen component, a place for gathering socially.	**✔**			
Is installed at floor level.	**✔**			**✔**
Stains, smudges, throws sparks.		**✔**		**✔**
Wastes firewood.		**✔**		
Emits large quantities of smoke.		**✔**		**✔**
Produces intense fire—is dangerous for children.		**✔**		**✔**
Causes burning eyes and throat.		**✔**		**✔**
**New improved cookstove: uses and advantages/benefits**				
Functions with a small amount of firewood.		**✔**		**✔**
Does not release smoke (smoke is not inhaled, does not hurt eyes).		**✔**		**✔**
Allows for cooking different foods simultaneously.		**✔**		**✔**
Keeps pots and pans clean.		**✔**		**✔**
Keeps food hot.		**✔**		**✔**
Reduces incidents of burnt hands and child burns.		**✔**		**✔**
Does not make women and children sick.		**✔**		**✔**
Improves the taste of food.				**✔**
Is identified with the Patsari improved stove program.				**✔**
Does not require consent from husband.				**✔**
Is installed at high level (not at floor level).	**✔**			**✔**
**Reasons for abandoning the improved cookstove**				
Kitchen was moved to a different place	**✔**			
Stove was installed in a different place in the kitchen.	**✔**			
User does not live in the household.			**✔**	
Stove uses a specific type of firewood.	**✔**			**✔**
Stove broke down and was not repaired.	**✔**		**✔**	
**Reasons for dismantling the improved cookstove**				
Stove design was unsatisfactory.	**✔**			
Stove was made of low-quality materials.	**✔**			
Kitchen was moved to a different place.	**✔**			
User moved.	**✔**			
Family was extended.	**✔**			
Family was nuclear.				**✔**
Husband or in-laws had an adverse opinion of the stove.	**✔**			
Program follow-up was not implemented.	**✔**			
Program was implemented by men.	**✔**			
**Wood smoke as a health risk**				
Were aware of health damages caused by the traditional stove (but were not certain that they would be affected).	**✔**			
Were aware of health damages caused by the traditional open fire (were certain that wood smoke caused disease and death).				**✔**
Had a high-risk perception of wood smoke exposure.				**✔**
Believed that use of the improved stove reduced wood smoke exposure and health risks, and therefore contributed to greater individual, family and collective well-being.				**✔**

### Women, the traditional stove, the *nixtamal* and *tortillas* (gender roles)

Adoption and use of traditional over improved stoves were largely conditional on gender roles. As documented in the previous section on everyday life, the indigenous sample communities regarded all domestic tasks, particularly cooking and related activities, as the responsibility of women. The respondents related that, in order to fulfill the norms and expectations of Purepecha society, women taught their daughters how to light the traditional open fire, prepare the *nixtamal* and make *tortillas* from an early age (between eight and ten years). These tasks were considered of great importance from a domestic viewpoint. Girls progressively acquired the knowledge and skills specific to their gender role and learned to play their wife-mother role as a fundamental condition for getting married (which, according to reports, occurred between the ages of 13 and 14 years).

“*She’d tell me [referring to her mother], ‘dear, learn to make* tortillas, *because one day you’ll get married and you won’t know anything about making* tortillas. *You’ll get scolded if you don’t know.’ So I used to help her, like that, little by little (one step at a time) and I learned.”*

*(female, 36 years old, ICS nonexistent, Quinceo*)

Women who did not acquire this knowledge and did not assume this role faced social disapproval, particularly by their husbands and in-laws. In the words of a participant:

“*The mothers-in-law are angry all the time and say that you don’t know how to do anything. Sometimes they even say (they tell the husband), ‘Get rid of that woman. What do I want her around here for?*”

(36-year-old woman, ICS nonexistent, Quinceo).

The early socialization about the traditional cooking practices using an open fire limits the adoption and use of the ICS.

Gender in these communities implies a series of man-woman inequalities reflected in decision making, for instance, in the adoption of new technology. Within the scope of our study, the majority of indigenous women were required to ask their husbands or in-laws for permission to participate in the Patsari ICS program. In some cases, a negative opinion from their male relatives was decisive in abandoning or destroying the stove.

Furthermore, men demanded that women use the traditional open fire for daily cooking. Being the principal household providers, responsible for the agricultural and woodworking activities in the community, their opinion was conclusive:

“*My dad comes and says, ‘Give them something to eat, quick (referring to the wood workers)…’ yeah, that’s why we do it here (on the traditional open fire) or over there, on the other traditional stove, real fast*”

(*35-year-old woman, ICS nonexistent, Quinceo*)

Another significant factor behind technology uptake was the fact that several steps of the program for example stove manufacturing and maintenance was implemented by men. The respondents reported that, owing to Purepecha customs and traditions, they felt uncomfortable when a man entered their homes while they were alone, and anticipated a potential conflict with their husbands.

In the mestizo community, however, gender relations were expressed differently: the majority of women accepted ICS installation on their own, that is, without needing the consent of their husbands. In some cases, the husbands and/or in-laws even encouraged the women to enroll in the program and advised them to stay calm and patient while learning how to operate the new stove. Although women in this community were also required to learn how to perform domestic tasks from a very early age, none of them mentioned that being able to use the traditional open fire was a fundamental part of their knowledge. In fact, respondents from this community related that, when they were children, the traditional three-stone or floor-level stoves were already obsolete and had been replaced by raised stoves, often mounted on a metal drum referred to as a “*tambo”*.

### Uses, advantages and disadvantages of the traditional stove

Although all of the sample women were using traditional open fire at the time of the study, usage varied according to their ICS status. Some women who had begun using the ICS continued using their traditional stove for preparing the *nixtamal* (once or twice a week), for heating bathwater for the family and for making *tortillas* during the planting and harvesting seasons. They also preferred the traditional stove for preparing food in a hurry or under pressure.

“*I use the big stove to make pozole, sometimes [to prepare] the nixtamal and sometimes when it’s very cold. But I use the chimney stove to cook and to make tortillas. When there’s a need to make pozole, when my girls have a birthday or want to invite friends over to eat, I do it down there. Or when there’s corn you have to roast it and you have to do it down there. You can’t do it at the chimney stove… It’s not every day. It’s almost only once a week.*”

(*29-year-old female, ICS in use, Quinceo*)

The women who had stopped using or had dismantled the ICS, used their traditional stoves for all their cooking activities, to heat the bathwater for the family and water to wash the dishes.

All the indigenous women in our sample, whether or not they still kept and were using the ICS, commonly resorted to the traditional stove for making large volumes of *tortillas* or food in pans, vats, pots or drums. Extensive cooking took place during traditional community celebrations and/or family events such as weddings, 15th birthday celebration and other birthdays, religious festivities and graduations. In one single day of celebration, they may prepare food for over 200 guests in only one house. Preparation was carried out by over 30 women using as many as six floor-level open fires. Respondents explained that some *comales* (a clay or metal disk used for cooking corn tortillas and for toasting coffee beans or cocoa) were so large that five women could use them simultaneously for making *tortillas*. The ICS was not viable for those cooking practices.

All of the indigenous women, whether or not they had kept and were using the improved stove, remarked that the traditional open fire generated intense heat, could be loaded with abundant firewood and allowed for cooking and making *tortillas* more rapidly than the ICS. The “speed” and “practicality” of the traditional stove were of primary importance for extended families needing to prepare food for many people or to make breakfast very early in the day for schoolchildren, husbands or other relatives working in the farm fields or wood sites.

Because of these characteristics, the traditional stove was highly valued and regarded as an indispensable asset by the indigenous women in the sample.

*Interpreter: “Yes, the lady decided to dismantle it [the ICS]. Down there you can cook faster, even though there’s smoke and the flame is big. The woman says the traditional stove is better than the Patsari. She says that’s why, because it has a bigger flame. That’s why it’s more valuable than the Patsari.*”

(*26-year-old woman, ICS nonexistent, Turícuaro*)

Furthermore, they commented that the traditional open fire could be lit with green firewood and logs of all sizes, a big advantage to them. In general, the fire was regarded as an important part of the kitchen, the space most commonly used for socializing. All family members can gather around the stove, sitting directly on the floor or on small wooden chairs.

Likewise, the women commented that making *tortillas* while sitting or kneeling—an activity which they referred to as *tortear*–made them feel as if they were resting, and allowed them to exert more strength while regrinding the dough—a feeling completely different from performing the same activity while standing. They reported being accustomed to the traditional stove at floor level because many of their daily activities were performed in a sitting or kneeling position: regrinding dough, making *tortillas*, preparing *atole* (a thin porridge-like drink), cooking, grinding chili peppers, doing the dishes and washing clothes. One of the respondents suggested that the ICSs should be installed lower, at floor level.

“… *when we finally sit down, we rest a little, for a little while. Here, we get tired of standing… Sometimes it feels heavy on the knees and here. Over there we sit for a little while and make tortillas*.”

(*32-year-old female, ICS abandoned, Quinceo*)

Even though the indigenous respondents saw numerous advantages in the traditional stove, they also recognized certain characteristics that they either disliked or perceived as disadvantages. ICS users mentioned that the traditional stove stained and smudged their hands and dishes, used an excessive amount of firewood and produced a great deal of smoke, thereby smoking up the entire kitchen and damaging or leaving their clothing dingy. Moreover they mentioned that the open stove emitted sparks everywhere, representing a danger for the children and for themselves. They also related that the smoke caused a burning of the eyes and a sore throat.

Within the group of women who had either abandoned the use of the ICS or had disposed of it altogether, there were those who recognized the traditional stove as a source of danger exposing users to accidents and noxious smoke. Nonetheless, while concerned about these things, they felt that the traditional stove was preferable simply for the tremendous heat it generated. Some participants saw no disadvantages whatsoever to traditional technology.

Although women in the mestizo community were familiar with the traditional open fire, they no longer used it in their daily cooking, with eight of them recurring to it very few times a year: at family celebrations and in the community festival. Only two participants continued to use it occasionally for heating bathwater and for preparing the *nixtamal*.

Unlike the indigenous women, mestizo women commented that they saw no advantage in using the traditional stove, observing moreover that woodsmoke caused headaches and irritation of the eyes, and that the sparks from the stove could lead to burns.

### Uses and advantages/benefits of the ICS

In the indigenous communities, eight of the nine ICS users employed the stove on a daily basis to prepare meals, five also used it to make *tortillas*, two to prepare the *nixtamal* and beans, and one to heat water. Among the multiple advantages associated by respondents with the new technology, the ones most frequently cited concerned ICS design: it required less firewood, did not produce smoke in the kitchen (a user from Quinceo even compared it with the gas stove), allowed for performing a number of tasks simultaneously (e.g., cooking meals and making *tortillas*), did not dirty or blacken pots, and remained hot for a long time, keeping the food hot until the moment it was served:

“*You can prepare meals and tortillas at the same time and they [the ISCs] heat quickly and use less firewood. And the advantage is that the smoke no longer comes into the kitchen. Now the smoke goes directly outside. There’s no more smoke. If you´re cooking, it’s as if you were cooking with a gas stove. There’s no smoke, and if there is smoke, it’s very little*.”

(*29-year-old woman, ICS nonexistent, Quinceo*)

Regarding health effects, ICS users from the indigenous communities reported no longer breathing smoke, suffering from watery eyes, burning their hands while making *tortillas*, or worrying about their children getting burned.

Those with abandoned or nonexistent ICSs still acknowledged some advantages of the new technology principally that it eliminated exposure to smoke.

The ICS users in the mestizo community commended the advantages of the new technology even more than did the indigenous users. Most frequently, they mentioned that it could be used for almost all of their daily activities: to cook meals, prepare the *nixtamal* and heat water for bathing; in their own words: *“We use it for everything*.*”* This multiplicity of functions led mestizo women to view their new stoves as a *“necessary or indispensable”* asset. They commented that the ICS cooked food more quickly, allowed for preparing several dishes simultaneously, maintained its heat for a long time allowing the food to stay warm until serving time, used less firewood than the traditional stoves, did not expose children to accidents, did not dirty their clothes while they cooked or made *tortillas*, improved the flavor of the food, as it no longer tasted like smoke, and prevented respiratory diseases in the children and in themselves. They highlighted that ICS use entailed health benefits for the entire family because the units were enclosed and people no longer inhaled smoke.

“*I think it helps us [women] as much as it does the family and the children, because in fact there was a lot of smoke with the ‘tambo’ and we absorbed as much of it as the children and everyone else, because we were always shut up inside*.”

(*29-year-old woman, ICS in use, La Mojonera*)

In some cases, women referred to the Patsari implementation program as “my program,” arguing that some family members had “become very fond of” the new stove. One of the respondents mentioned that her daughter referred to the stove as “my tongue” owing to the small size of the flame. Respondents commented that the stove had brought them many benefits, and their daily lives had changed significantly as a result of using the Patsari ICS. As reported by the La Mojonera health center nurse, 90% of the 464 families in the community had adopted an ICS from the Patsari or another program and those who had not obtained a Patsari either desired or were looking for one.

### Reasons for abandoning use of the ICS

In the indigenous communities, two participants reported having changed the location of their kitchens: one because the previous space was insufficient for their growing family; the other because they joined the aforementioned “Piso firme” program. In both cases, their ICSs had been exposed to the elements and could no longer be used. In other cases, the ICS had been installed somewhere other than the kitchen. Finally, two participants commented that they were not using the new stove because they had no dry firewood or adequate-sized logs. They also noted that the ICS griddles were deteriorating—the iron was peeling and adhering to the *tortillas—*and the stove could not be used for preparing large amounts of food.

In the mestizo community all the installed ICS were present and in use except for two abandoned stoves. One of the two women who had abandoned use of the ICS explained that she no longer lived where the stove had been installed; the other one reported that the stove had broken and she had been unable to repair it.

### Reasons for dismantling or disposing of the ICS

Likewise, the indigenous women who no longer used the stove (22) adduced numerous reasons for this, many of them interrelated. Some mentioned that the wooden base had burned or rotted or the flame tended to go out causing a great deal of smoke. This had led them to remove the stove from the kitchen or abandon its use to the point where it was ruined. Others mentioned that rain had entered through the stovepipe and had destroyed the stove, or the kitchen roof had fallen in and had crushed the stove. In other cases the respondents felt that the stove did not provide sufficient heat for cooking, leading them to progressively widen the combustion chamber until the stove was completely destroyed.

Additional reasons pertained to household/community characteristics. Six respondents reported having removed the ICS to make room for a bedroom; four destroyed the stove because they moved from their in-laws’ house to their own in order to leave the space for another member of the family (a man who had just gotten married). Others moved the kitchen to another location within the house, either because the family had grown or simply because they no longer liked the former location for the kitchen. In another case, the father-in-law destroyed the ICS of a couple who had migrated to the United States to work.

All three groups pointed to issues concerning ICS program implementation. Some respondents considered the follow-up as inadequate, and didn’t know where to turn to when they had questions about the functioning of the stove or when it needed spare parts.

It is important to bear in mind that various factors may influence the decision to dismantle or abandon use of the ICS. For example one participant from Quinceo did not know how to light the stove: the flame would die out and produce a great deal of smoke; she had neither small pieces of firewood nor a hatchet with which to cut the logs. In this case, both the ICS and the traditional stove were in the kitchen and, because the family needed to use that space as a bedroom, the husband of the participant decided to do away with the ICS arguing that it didn’t work properly.

### Woodsmoke as a health risk

When the indigenous ICS users were asked if woodsmoke could damage health, they responded that smoke could definitely have an effect on health, that it could cause coughing and hurt the lungs or respiratory tract. They emphasized that women and children were the most affected because they spent the most time in the kitchen. One of the participants mentioned that her father-in-law had died from the effects of woodsmoke, while another mentioned knowing people who had died of lung cancer as a result of cooking with firewood.

“*I feel that yes, mostly for this reason….through the airways, lung infection and all of that….like, for example, when…there are a lot of people who smoke, their lungs are affected for the same reason, because smoke gets into them. I say it’s the same with firewood, right?*”

(*26-year-old woman, ICS in use, Turicuaro*).

Among the group of women who had abandoned use or disposed of the ICSs, some were aware that smoke could damage the lungs but were unsure whether they themselves could become ill. Even knowing that the traditional stove could cause harm, they lit it because it was quicker for cooking and because they couldn’t “do everything” on the Patsari. One of the participants was aware that smoke was harmful to health, but noted that the decision to stop using the stove did not depend on her. Women who had disposed of the ICS mentioned that they had received information about harm to health from the community clinic and from the people who made the improved cookstove.

In the mestizo community, all the women agreed that woodsmoke could affect health. They had learned this in part through the community health center, but also from knowing family members or acquaintances who had lung diseases as a result of exposure to woodsmoke. The respondents used phrases such as “s/he has smoke in the lungs,” “smoke plugs the lungs” or “their lungs are damaged,” and compared woodsmoke to cigarette smoke in terms of harm. They perceived woodsmoke as a risk to which they had been exposed since birth, and could even identify diseases such as cancer and COPD. All the women agreed that they were more vulnerable to damage from smoke as a result of cooking on the traditional stove.

“…*since birth, you’re exposed to smoke…because, well, from the time you’re born, your parents pull you near the fire or the stove. Before, well, there were none of these burners. There’s a neighbor, her lungs are already really blocked and she already receives oxygen. At night she uses it. And I also know another woman, a sister of my mother-in-law, she also gets oxygen at night because of all the smoke she breathed her whole life, and her lungs are already damaged*…”

(*38-year-old woman, ICS in use, La Mojonera*)

Perceived ICS benefits related mainly to matters of health: “You don’t inhale smoke anymore;” “It helps protect the family’s health, especially the children;” and “It prevents respiratory diseases like the flu or cough.” The women felt that they were particularly at risk because they spent more time in the kitchen than did the men. They had a high-risk perception of woodsmoke based on the following link: ICS = less exposure to smoke = lower health risk.

## Discussion

In analyzing the three study communities, it became evident that very few women in indigenous communities used ICSs and the majority ended up dismantling them. In the mestizo community, on the other hand, the majority of women maintained and used ICSs for cooking and other domestic tasks.

A variety of factors have important implications which serve to either enable or limit the use of the new technology in both indigenous and mestizo communities: family characteristics, certain aspects of women’s daily lives, gender relations, sociocultural factors associated with the traditional open fire, ICS issues with design as well as program implementation and follow-up, and social perceptions on exposure to smoke.

In indigenous communities, the extended family and certain daily activities such as making *tortillas*, sometimes as often as twice a day, work against the use of ICSs. In these communities, women prefer traditional open fires considered better due to the amount of food that women must prepare every day and, the speed and functionality they require to be able to both cook and perform other numerous activities. It is important to note that both characteristics, the extended family and the daily lives of the women, are rooted in a set of local beliefs and sociocultural norms that govern Purepecha community life. In their culture, newlyweds usually remain in the house of the parents because they tend to marry very young and parents prefer to keep them near until they are capable of forming their own home. Purepechas also believe that newly married couples who build their own house immediately after the wedding will have a short marriage [48].

Although the daily activities of mestizo women resemble those of their indigenous counterparts, with domestic work assigned to women in both cases, cooking on a traditional open fire is no longer “obligatory”. Some mestizo women have never used a traditional stove, not even in their childhood. The fact that the mestizo community has better sociodemographic indicators as described previously, compared with the other two, allows us to partly understand the results. For example, as La Mojonera has less overcrowded households, with availability of more spaces (greater number of rooms) allows the kitchen to be thought of as an independent place where a specific object can be located like the stove. The educational level also translates into greater access to information by women, and the possession of goods such as the refrigerator allows women to make tortillas only every third or fourth day, as one of the participants says:

"*every third day I make tortillas*,… *in the morning I make them. Then I just put them together and wrap them in a napkin and in a bag and put them in the refrigerator*

(*37-year-old woman, ICS in use, La Mojonera*).

The sociodemographic context favors the knowledge about the stoves and their benefits promoting the use of the ICS. In addition, fewer people reside in mestizo compared to indigenous households and therefore require less food preparation. Our results are in line with those of other studies which have demonstrated the impact of environment and family composition on the adoption and use of ICSs. For instance, a large number of adults and economically active individuals in a household has been described as a factor that discourages the use of ICSs by Inayatullah (2012) [17] in Pakistani rural communities and by Adrianzen (2009) in Peru [49], while having a woman as head of household favors the uptake of new technology [49, 50], because they are the main beneficiaries. In these communities, the women are responsible for collecting firewood and spend most of their time inside the household, therefore they are more aware of indoor pollution; moreover they are also responsible for the health of children and the elderly. For these reasons, the ICSs are more likely to be adopted in order to reduce the incidence of respiratory diseases when a woman is the head of household.

The characteristics and location of households are also factors that determine the use of improved technologies. According to our study, the structure of the house, the constant rearranging of household space according to family dynamics, the use of the kitchen as a bedroom and even the climate were contributing factors to abandoning use of the Patsari ICS. Similar results have been reported by other studies where household characteristics, geographical location and the climate have proved crucial for the sustained use of ICSs [4, 24, 26, 49, 51, 52]. For example, the study by Troncoso et al. (2007) in Mexico reported that the ICS helped keep houses fresher [4], advantageous in summer or warm climates.

In the indigenous and rural context, the domestic chores of young wives are directed and supervised by their husbands and their in-laws, reducing housewive´s autonomy to make decisions on how to perform their daily activities. Social expectations about “being a woman” played a key role in the possibility of incorporating and using new technologies and determined to a great extent what was/was not possible in a society where women play defined roles. The sway of the traditional open fire among the indigenous women in our study formed part of those expectations. In a study in Kenya [26] women were also unable to make decisions on cooking technology because all decisions were contingent upon the opinion of the husbands and older wives. In this community, families were extended because men are allowed to have several women, but it was not mentioned whether or not traditional technology was more appropriate for preparing large amounts of food, as reported in our study. Another study of Sudan noted that the relationship between income and adoption of new technology was influenced by the patriarchal structure, as women received only part of the family income to buy fuel or an ICS [18].

Use of the traditional open fire by the Purepecha women for preparing large quantities of traditional dishes during family celebrations, social events and festivals that draw the entire community for religious or patronal feasts should not be viewed as an isolated social practice, but as part of the foundations of the Purepecha culture or as traditions and customs that shape the Purepecha identity. These festivals, besides food, feature fireworks, varied ceremonies and rituals, music and dance, renewal of faith, religious expression, sacred acts, and exaltation of community identity [53]. Use of the traditional stove and cooking practices reaffirm the Purepecha identity as an ethnic group, fulfill important sociocultural functions [18] and carry an intrinsic value among these groups [16]. At the same time, they render adoption of new cooking technologies difficult.

Conversely, the majority of mestizo women cook on the Patsari ICS and see no advantage to using the traditional open fire. One of the factors facilitating the adoption of new technology in these communities is the predominance of nuclear families, with women no longer needing to make *tortillas* every day. Mestizo women ascribe many advantages to the Patsari stove, particularly to its design. According to studies in India, Mongolia and Kenya, one of the advantages of using improved cooking technologies is that they maintain cleanliness in the kitchen and the house [25, 27, 54]. Mestizo women participate in forms of social organization and have daily lives that are different from those of their indigenous counterparts. They are not particularly attached to the traditional stove and do not associate it with deeply rooted traditions.

The social organization of a community as well as the type and intensity of relations among its residents—defined as social capital in some studies—can also affect the process of change and the adoption of new technology. A study in Peru demonstrated that social capital can have positive or negative effects on the adoption of ICSs. For example, if many families report problems with the new stoves and this information is disseminated through a powerful social network, the impact on adoption of the stove can be negative. ICSs also tend to be rejected or fall into disuse if favorable information does not spread throughout the community owing to weak social links [49]. Although we did not use an instrument to measure social capital, our thematic content analysis confirmed that social interrelations within a community can help or hinder the adoption and use of improved cooking technologies.

It should also be noted that the design of ICSs can either favor or restrain their adoption, depending on the characteristics of the community. For example, in indigenous communities, the height of the raised stove, is an impediment, given that the women usually carry out their activities seated for greater comfort. On the other hand, height favors adoption among mestizo communities given the previous experience of the women with raised stoves. This factor has already been documented in Mexico by Romieu et al. (2009), Troncoso et al. (2007) and other authors. In other countries too design factors have been found to affect the adoption and continued use of new technologies. One recurring problem in uptake has been that ICSs require small pieces of firewood not always available in these homes [15, 24, 27].

Insufficient user training and lack of program follow-up are factors that also contribute to rendering use of ICSs more difficult. Studies indicate that the complexity of use is one of the disadvantages of improved technology. Similarly, issues related to accessibility, repair and maintenance are decisive in the use or abandonment of ICSs [4, 55].

Although indigenous women normally discuss the potential damage to health of traditional stoves, they are uncertain as to whether they themselves will be affected in the future and their frequent allusions to the great value of the traditional open fire indicate that the issue of risk is mostly a matter of talk originating outside the community. From a cultural perspective, it can be observed that definitions of risk and danger are based on concepts and interpretations rooted in society as a result of sociocultural processes, and not as an isolated phenomenon. Hence, what is understood as polluted, and therefore dangerous, is that which threatens the social order, the integrity of one’s body or the symbolic body of the community or society to which one belongs [56]. The degree of exposure to woodsmoke is thus influenced by sociocultural factors more than by lack of information [57]. Public health personnel and institutions should play a key role in designing campaigns and educational programs with participatory approaches to discuss the acute and chronic effects of indoor air pollution with the population. To do that, these personnel should be trained to work within intercultural contexts to participate in a longer follow up intervention programs. Since acute respiratory diseases is the main cause of medical demand in these communities, a major contribution from the health sector is expected during home visits as well as during the visits to clinics.

Fire plays a central role in Purepecha´s view of the world and the origin of the universe, and therefore neither fire nor the smoke that comes from it can be considered elements that damage health; on the contrary, according to the worldview of these indigenous communities, they are part of the life principle [58]. Similarly, rural women from Bangladesh did not perceive indoor air pollution as a significant health risk and prioritized other basic development needs (e.g., good schools, sanitary bathrooms and free medical consultation) over the ICS. Considerations unrelated to health also dominate decision making in the home, thus limiting the adoption of new technology [59].

By contrast, in the mestizo community, women have established a link between the use of ICSs and wellbeing: less exposure to woodsmoke = less risk to health = greater wellbeing of the individual and the collective. The benefits of not breathing smoke, associated with improved health, have also constituted one of the qualities recognized by ICS users at the international level [24, 26, 27, 60]. Information about health risks is a factor that has been frequently associated with the adoption and sustained use of new technology [17, 61].

We do not think that ICS implementation programs should be abandoned, but it is necessary to design and offer a portfolio of alternatives for the diverse household energy uses in order to displace the polluting open fire, including the use of cleaner technologies and fuels as LPG, biogas and solar energy, as proposed by the WHO [62]. For example, Nicaragua, Honduras and Peru have national biomass stove programs and are conducting intervention efforts together with international agencies and locally based NGOs. Peru has installed a government certification laboratory for biomass stoves establishing minimum performance standards, and in addition, evaluates and certifies improved kitchens, and offers brief courses on constructing models for certified improved kitchens. The Peruvian government program for distributing improved stoves is registered with the Gold Standard organization in order to receive carbon credits. In addition, its Universal Plan for Energy Access is oriented towards expanding the energy frontier to include the most vulnerable population by means of mass production of natural gas based on renewable energy and the promotion of LPG access among urban and rural sectors [63].

It is necessary to bear in mind that the ongoing global discussions on domestic fuels have proceeded beyond kerosene, LPG, and biogas, focusing now on latest generation bio combustibles (ethanol and methanol) which could become valuable options for ensuring healthy cooking and a respect for the environment [62] providing those fuels are available.

New technologies will be accepted by the population if their views and needs are considered. An enabling environment, can only be provided by the cooperation of stakeholders at various levels and proper government leadership in formulating policies and domestic energy programs based on user demand and perceptions [8].

## Conclusions

The most important recommendation arising from this study is therefore to ensure that ICS implementation strategies are tailored to address the specific—and often very different—needs and ethnographic characteristics of both the indigenous and mestizo communities. These characteristics include social organization, gender roles, beliefs regarding the use of the traditional stove, everyday life, particularly that of women, popular cooking practices, traditions and social perceptions on exposure to woodsmoke. Nuclear families were more receptive to new technology, and ICS implementation in extended families should take into account the number of people living in a house as an indicator of the number of stoves required. Furthermore, ICS programs could be part of an overall improvement of the dwelling, including the household floor, bathroom, stove (as it appears in the guidelines of the Mexican Ministry of Agrarian, Land, and Urban Development—SEDATU) and heating in cold areas.

Men should be incorporated into the implementation of these technologies to mitigate gender relations of power and subordination that render technological change, including ICS use, more difficult to achieve. In particular, women should take easier decisions within the home, and their interests should be heard and made more visible.

ICS programs should offer a number of alternatives such that women and their families can choose the option best suited to their needs, providing all reduce pollution and improved health is expected. ICS programs must include funding for proper training and communication, maintenance, spare parts or repairs and adequate follow-up with indicators incorporating users’ opinions. Proper knowledge of the adverse impact that smoke from traditional woodstoves and open fires can exert on health and the environment is key, as the ICS implementation is a derived solution. Implementation of this type of strategy must be carried out carefully and gradually; an abrupt substitution of traditional practices only generates greater resistance to change. Thus, a portfolio of options will be needed to satisfy all cooking tasks and other household energy needs.
